# Flexible N-Type Thermoelectric Composites Based on Non-Conductive Polymer with Innovative Bi_2_Se_3_-CNT Hybrid Nanostructured Filler

**DOI:** 10.3390/polym13234264

**Published:** 2021-12-06

**Authors:** Juris Bitenieks, Krisjanis Buks, Remo Merijs-Meri, Jana Andzane, Tatjana Ivanova, Lasma Bugovecka, Vanda Voikiva, Janis Zicans, Donats Erts

**Affiliations:** 1 Institute of Polymer Materials, Faculty of Materials Science and Applied Chemistry, LV-1048 Riga, Latvia; juris.bitenieks@rtu.lv (J.B.); remo.merijs-meri@rtu.lv (R.M.-M.); tatjana.ivanova@rtu.lv (T.I.); janis.zicans@rtu.lv (J.Z.); 2 Institute of Chemical Physics, University of Latvia, LV-1586 Riga, Latvia; krisjanis.buks@lu.lv (K.B.); jana.andzane@lu.lv (J.A.); lasma.bugovecka@lu.lv (L.B.); vanda.voikiva@lu.lv (V.V.)

**Keywords:** flexible thermoelectric composites, hybrid network, polyvinyl alcohol based composite materials, carbon nanotubes, bismuth selenide nanostructures

## Abstract

This research is devoted to the fabrication of polyvinyl alcohol (PVOH) based n-type thermoelectric composites with innovative hybrid bismuth selenide-multiwalled carbon nanotube (Bi_2_Se_3_-MWCNT) fillers for application in flexible thermoelectric devices. Hybrid fillers were synthesized by direct deposition of Bi_2_Se_3_ on multiwalled carbon nanotubes using a physical vapor deposition method, thus ensuring direct electrical contact between the carbon nanotubes and Bi_2_Se_3_. The Seebeck coefficient of prepared PVOH/Bi_2_Se_3_-MWCNT composites was found to be comparable with that for the Bi_2_Se_3_ thin films, reaching −100 µV·K^−1^ for the composite with 30 wt.% filler, and fluctuations of the resistance of these composites did not exceed 1% during 100 repetitive bending cycles down to 10 mm radius, indicating the good mechanical durability of these composites and proving their high potential for application in flexible thermoelectrics. In addition, other properties of the fabricated composites that are important for the use of polymer-based materials such as thermal stability, storage modulus and linear coefficient of thermal expansion were found to be improved in comparison with the neat PVOH.

## 1. Introduction

Thermoelectric materials and technologies based on them such as thermoelectrical generators (TEG) enable the direct conversion of heat to electricity. Such devices are expected to play an important role in solving one of the biggest global challenges, which is an increase of energy efficiency of domestic and industrial processes by waste heat capturing and conversion to useful energy. In the field of TEGs, the development of flexible devices is an emerging trend. It is motivated by the variety of heated surfaces not having linear shapes as in, for instance, hot pipes. Another motivation for development of flexible portable TEGs is the need for wearable and autonomous devices. Personal electronic devices (including biometric monitoring) are common in daily lives and often rely on batteries. Replacement of battery-powered wearable devices with TEGs operating at the temperature difference between the human body and surrounding environment would significantly reduce the need in small batteries for such devices and thus contribute to the reduction of environmental contamination.

The best thermoelectric materials for thermoelectrical applications at temperatures close to room temperature (from −50 up to 200 °C) are inorganic bismuth and antimony chalcogenides (Bi_2_Se_3_, Bi_2_Te_3_, Sb_2_Te_3_) [1].

TE materials are characterized by a dimensionless figure of merit ZT, defined as S^2^_ϭ_T/k, where S is Seebeck coefficient of the material, _ϭ_ is its electrical conductivity, T is absolute temperature and k is thermal conductivity of the material. S^2^_ϭ_ is usually referred to as power factor (PF) of thermoelectric material. The common approaches in enhancement of ZT are maximizing PF and/or reduction of thermal conductivity of the material by different approaches such as alloying, nanostructuring, introduction of defects, and doping [1,2]. The use of bulk inorganic materials in flexible TE devices is precluded by their inherent rigidity, and the research related to the flexible TEGs was mostly focused on electrically conductive polymer TE materials and these materials-based composites [3]. Regarding n-type materials, some of the more promising polymeric n-type semiconductors include naphtalenedicarboximide bithiophene derivatives, linear ladder type polymers, halogenated derivatives of benzodifurandione poly(p-phenylene vinylene) and insoluble organometallic compounds as reported by Beretta et al. [4]. However, the significant drawback of these materials is their poor stability in air and humid environment and very narrow range of operation temperatures limited to near-room values [5].

Besides already known chalcogenides properties of the p- and n-type conducting thermoelectric materials may be improved with various metallic [6], metal oxide [7] as well as carbon based materials like graphene [8], carbon fibers [9] and carbon nanotubes (CNTs) [10,11]. In CNT based polymer-CNT composite materials the CNTs within the polymer matrix form electrically but not thermally conductive networks, thus improving thermoelectrical ZT of the composite, as well as their thermal stability and mechanical properties [12,13]. However, fabrication of thermoelectric composites based on n-type electrically conductive polymers requires pre-annealing of CNT filler in vacuum to desorb oxygen and convert the CNT conductivity type from their natural p-type to n-type [14], which further degrades due to the exposure of the composite to air, resulting in a decrease of its PF [13]. The best reported ZT values obtained for the CNT-polymer composites were still too low for commercialization [10,15]. Fabrication of inorganic/organic hybrids, where electrically conductive polymer is used as a matrix for supporting a network of inorganic TE nanostructures such as flakes, nanoplates or nanowires to take advantages of the properties of conducting polymers and inorganic nanostructures, also did not result in ZTs comparable with the ZTs of bare inorganic materials due to the uneven dispersion of inorganic nanostructures, as well as the mismatch between the Fermi energy levels of inorganic nanostructures and conducting polymer, resulting in energy barriers for charge carrier transfer between the inorganic and organic phases.

Recently, Pires et al. [16] demonstrated alternative approach for fabrication of flexible TE composites, which is based on the use of non-conductive polymer as a matrix for thermoelectric filler. In this approach, electrically non-conductive environmentally friendly polyvinyl alcohol (PVOH) mixed with very high concentration (60–90 wt.%) of Bi_2_Te_3_ micro-particles to fabricate PVOH/Bi_2_Te_3_ n-type TE composites. These PVOH/Bi_2_Te_3_ composites were reported to have a Seebeck coefficient comparable with the properties of Bi_2_Te_3_ microparticles, however, very high electrical resistance, resulting in low PF of the composites, except for the composite with 90 wt.% of the Bi_2_Te_3_ filler, which requires a significant amount of rare and toxic filler.

In this work, effective n-type thermoelectric composites were prepared by mixing PVOH with innovative bismuth selenide-multiwalled carbon nanotube (Bi_2_Se_3_-MWCNT) hybrid networks fabricated by simple catalyst-free physical vapor deposition method developed by our group [17,18,19,20,21]. Our group demonstrated that, despite the natural p-type of the as-grown MWCNTs, direct deposition of Bi_2_Se_3_ on the MWCNT network results in formation core-shell MWCNT-Bi_2_Se_3_ structures, supporting an interconnected network of Bi_2_Se_3_ nanostructures [17]. Such Bi_2_Se_3_-MWCNT hybrid networks with MWCNT content below 20 wt.% exhibited stable in ambient conditions n-type conductivity and Seebeck coefficients, comparable with that of bulk Bi_2_Se_3_. This was attributed to the impact of charge carrier transfer and filtering at the Bi_2_Se_3_-MWCNT interfaces, and to the contribution of the Bi_2_Se_3_ nanostructured network to the thermoelectric efficiency of these hybrid structures. Using such hybrid structures instead of inorganic micro- and nanoparticles dramatically reduces the amount of filler required for fabrication of PVOH-based n-type composites due to the formation of an electrically conductive MWCNT network, supporting and interconnecting the inorganic nanostructures.

The obtained thermoelectric composite materials have been characterized in respect to structural, thermoelectric, thermal and mechanical properties to evaluate their potential use for the design of low-power thermoelectric devices at various configurations of thermoelectric elements. It is expected that these n-type semiconducting materials could be technically suitable for application in flexible thermoelectric devices as the n-type component.

## 2. Materials and Methods

PVOH (Celvol E 04/88S) with the degree of hydrolysis of 88% (Celanese Corporation, Irving, TX, USA) was used as a non-conducting polymer matrix for the development of thermoelectric composites with various carbon nanotube-based nanofillers, including commercial MWCNTs with an outer mean diameter of 13 nm, inner mean diameter of 4 nm, bulk density of 130–150 kg·m^−3^ and purity of 95% (Baytubes^®^ C150 P, kindly donated by Bayer MaterialScience, Covestro, Leverkusen, Germany), as well as laboratory synthesized hybrid networks of Bi_2_Se_3_-MWCNT (content of MWCNTs in the network 10 wt.%). To prepare PVOH based nanocomposites, PVOH was initially dissolved in a distilled water at 80 °C. Then a fixed amount of the chosen thermoelectric filler was dispersed in PVOH solution by the ultrasonic device UIS250V (Hielscher Ultrasonics GmbH, Teltow, Germany). To induce an efficient dispersion of the thermoelectric filler within the PVOH matrix, the sonication time was approximately 20 min. Then, the PVOH dispersions obtained were cooled down to room temperature and applied by a drop coating method on clean glass substrates for thermoelectric property and structure characterization. Free-standing films for mechanical, calorimetric, and thermogravimetric property characterization were obtained by solution casting on a flat polypropylene surface.

Bi_2_Se_3_ nanostructures were deposited on prefabricated surfaces by a spray-coating technique MWCNT substrate by catalyst-free physical vapour deposition method described elsewhere [18,19,22,23,24]. The mass fraction of the deposited Bi_2_Se_3_ material on the MWCNT networks was varied by the amount of the source material and deposition time. The wt.% of the MWCNTs in the Bi_2_Se_3_-MWCNT hybrid networks was determined by weighing the substrate components at each stage of the hybrid network preparation (glass, glass covered with MWCNTs, glass covered with the MWCNTs and Bi_2_Se_3_) using analytical scales (Precisa XR 205SM-DR, Precisa, Dietikon, Switzerland). After weighing the Bi_2_Se_3_-MWCNT hybrid structures were manually scraped off the glass substrates to a container using a standard stainless-steel scalpel blade No 11. For comparison of the results with the data published by Pires et al. [16], the volumetric percentage (vol.%) of the Bi_2_Te_3_ filler in PVOH/Bi_2_Te_3_ composites was recalculated to the weight percentage (wt.%).

Characterization of the samples was performed using a Hitachi S-4800 field-emission scanning electron microscope (Hitachi, Tokyo, Japan) equipped with an energy-dispersive X-ray diffraction analyser (Bruker XFlash Quad 5040, Billerica, MA, USA) and transmission electron microscope FEI Tecnai GF 20 (FEI Company, Hillsboro, OR, USA).

Thermoelectrical measurements were carried out at room temperature (300 K) under ambient conditions using a home-made device allowing bending of the samples and calibrated by Standard Reference Material 3451 (NIST, Gaithersburg, MD, USA) for a low-temperature Seebeck coefficient as described elsewhere [24]. Room-temperature electrical characterization was performed using Keithley 6430 Sub-Femtoamp Remote Source Meter (Cleveland, OH, USA). Low-temperature resistance measurements were performed using thermal transport option of the physical property measurement system DynaCool9T (Quantum Design, San Diego, CA, USA). For the low-temperature measurements, sample strips of approximate size 10 mm × 5 mm on a glass substrate were formed. Etching of sample surfaces with Ar ions was performed for 15 min using a Gatan Precision etching and coating system 682 (Gatan, Pleasanton, CA, USA), operating at 2.6 keV voltage and 150 μA current.

Thermogravimetric analysis (TGA) was carried out using TGA1/SF device (Mettler Toledo Inc., Cleveland, OH, USA). Mass loss was determined at temperature range of 25–800 °C at a heating rate of 10 °C/min. Each sample mass was ~10 mg and the measurements were performed under ambient conditions.

Differential scanning calorimetry (DSC) measurements were measured using Mettler Toledo DSC 1/200W equipment. Experiments for PVOH-based thermoelectric composites were performed in a nitrogen atmosphere within the following temperature regime: (1) first heating from −90 °C to 250 °C, (2) cooling from 250 °C to −90 °C, (3) second heating from −90 °C to 250 °C. Temperature change rate of 10 °C/min. was used for all the heating-cooling-heating stages.

Dynamic mechanical thermal properties were determined in tensile mode using dynamic mechanical thermal analyser Mettler Toledo DMA/SDTA861. For PVOH and the selected PVOH based thermoelectric composites the experiments were performed in the temperature range from −10 °C to +80 °C within a single heating stage. Dynamic mechanical thermal analysis was performed at a heating rate of 2 °C/min and frequency of 1 Hz.

Thermomechanical characteristics were determined in tensile mode using TMA/SDTA841 device (Mettler Toledo Inc.) within the temperature interval from −10 °C up to 25 °C, which is below the PVOH glass transition temperature. The mechanical load applied during the measurements was in the range of 0.03 N.

## 3. Results and Discussion

**Figure 1:**
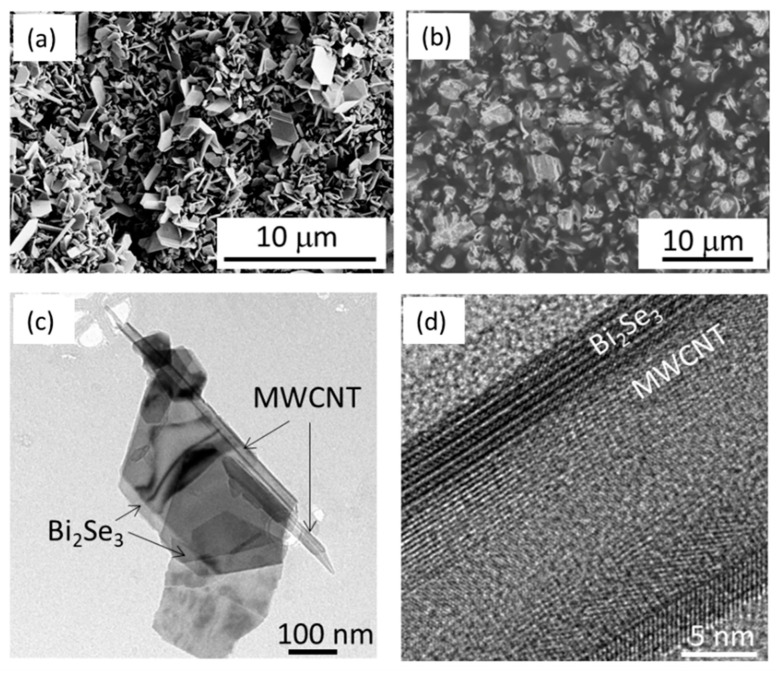
(**a**) Scanning electron microscopy (SEM) image of as-grown Bi_2_Se_3_-MWCNT (multiwalled carbon nanotube) hybrid structures with ratio 90 wt.% Bi_2_Se_3_: 10 wt.% MWCNT; (**b**) SEM image of the surface of polyvinyl alcohol (PVOH)-based composite with 30% Bi_2_Se_3_-MWCNT filler; (**c**) transmission electron microscope (TEM) image of a Bi_2_Se_3_-MWCNT hybrid structure; (**d**) High-resolution TEM image of Bi_2_Se_3_-MWCNT interface.

The Bi_2_Se_3_-MWCNT hybrid structures with ratio 90 wt.% Bi_2_Se_3_:10 wt.% MWCNT were (Figure 1a) used as a filler for preparation of PVOH-based composites with filler mass fractions 10 wt.%, 15 wt.%, 30 wt.% (Figure 1b) and 45 wt.%.

As previously reported by our group, the Bi_2_Se_3_-MWCNT nanostructures with 90 (Bi_2_Se_3_):10 (MWCNT) wt.% ratio showed n-type conductivity with the Seebeck coefficient ~−40 μV·K^−1^ [17], and it was expected that the presence of 10% of MWCNTs in the hybrid structures will ensure good enough electrical conductance without significant increase of its thermal conductance, when mixed together with the PVOH, as well as improve the mechanical properties of the polymer. The distribution of the Bi_2_Se_3_-MWCNT hybrid structures throughout the whole cross-section was checked by the measurements of the electrical conductivity of the composites, showing that both sides of the composite are electrically conductive. It was found that resistance of the samples containing 10 wt.% of Bi_2_Se_3_-MWCNT filler is beyond the upper measurement limit of the source meter (these samples may be considered to not be electrically conductive). In turn, the samples with 45 wt.% filler were too brittle to acquire free-standing films of decent quality. For these reasons, a further study was performed for the PVOH/Bi_2_Se_3_-MWCNT composites containing 15 and 30 wt.% of filler. Properties of these samples were compared with the properties of PVOH/Bi_2_Te_3_ composites with 68–90 wt.% of Bi_2_Te_3_ filler, reported by Pires et al. [16]. It should be noted the PVOH/Bi_2_Te_3_ composites reported by Pires et al. were printed on polyethylene terephthalate (PET) substrates, and only indicative photos were provided on their flexibility with no reference to filler wt.% and/or electrical stability vs. bending radius. There are no data available on the brittleness, flexibility, and electrical stability upon bending of free-standing PVOH/Bi_2_Te_3_ films. Table 1 summarizes the main properties of the prepared PVOH/Bi_2_Se_3_-MWCNT composites and the corresponding properties of the PVOH/Bi_2_Te_3_ composites with 68–90 wt.% of Bi_2_Te_3_ filler, reported by Pires et al. [16].

**Figure 2:**
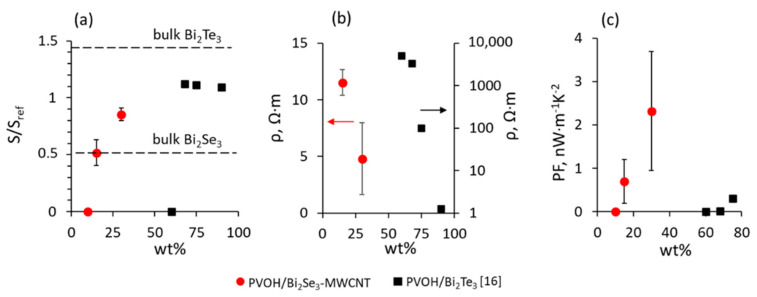
Comparison of the properties of the PVOH/Bi_2_Se_3_-MWCNT composites (red dots, this work) with the properties of PVOH/Bi_2_Te_3_ [16] (black squares) composites vs. filler wt.%: (**a**) Seebeck coefficient of the composite (S) relative to the Seebeck coefficient of the nanostructured materials (S_ref_) [16,22,26,27,28,29]; (**b**) resistivity; (**c**) power factor.

The averaged Seebeck coefficients of the PVOH/Bi_2_Se_3_-MWCNT composites were −60 μV·K^−1^ and to −100 μV·K^−1^ for 15 and 30 wt.% filler respectively (Table 1). Seebeck coefficient of the composite with 15 wt.% filler was comparable with that for bulk Bi_2_Se_3_ (~−68 μV·K^−1^ [25]), while the Seebeck coefficient of the composite with 30 wt.% filler was comparable with the reported Seebeck coefficients of Bi_2_Se_3_ nanostructured thin films (~−115 μV·K^−1^ [22,27,28,29]) and significantly exceeded the Seebeck coefficient of the bulk Bi_2_Se_3_. In contrast, in the work reported by Pires et al. [16], the PVOH/Bi_2_Te_3_ composites showed Seebeck coefficients of ~−173 μV·K^−1^ (Table 1), comparable with the Seebeck coefficients of nanostructured Bi_2_Te_3_ thin films (~−155 μV·K^−1^ [16]), only at filler concentrations exceeding 65 wt.%. However, these values were still lower in comparison with the Seebeck coefficient of the bulk Bi_2_Te_3_ (~−225 μV·K^−1^ [26]). Figure 2a illustrates the values of the Seebeck coefficient (S) of PVOH/Bi_2_Se_3_-MWCNT composites (red circles) and PVOH/Bi_2_Te_3_ composites (black squares) reported by Pires et al. [16], as well as values of the bulk Bi_2_Se_3_ and Bi_2_Te_3_ materials relative to the Seebeck coefficient values reported for the nanostructured Bi_2_Se_3_ or Bi_2_Te_3_ thin films (S_ref_). It should be noted that the values of the Seebeck coefficient shown by the PVOH/Bi_2_Se_3_-MWCNT composites were 1.5–2.5 times higher in comparison with the previously reported Seebeck coefficients of the bare Bi_2_Se_3_-MWCNT hybrid structures [17]. Presumably, this effect could be attributed to the influence of PVOH on the existing thermoelectric properties of material. Previously, the same effect of the increase of Seebeck coefficient by ~30% was reported for graphene and sodium cobalt oxide nanoparticles after their introduction into the PVOH matrix [30,31]. However, the mechanism of the increase of Seebeck coefficient upon introduction of the material into PVOH is not clarified yet.

The averaged resistivity of the PVOH/Bi_2_Se_3_-MWCNT with 15 wt.% and 30 wt.% Bi_2_Se_3_-MWCNT filler (11.5 Ω·m and 4.8 Ω·m respectively, Figure 2b, red dots, Table 1) was found to be up to 1–3 orders of magnitude lower in comparison with the data reported for PVOH/Bi_2_Te_3_ composites with 68–75 wt.% filler (10^2^–10^4^ Ω·m [16], Figure 2b, black squares, Table 1). Low resistance is presumably due to the MWCNT network formed in the polymer additional to the Bi_2_Se_3_ nanostructured network, and due to the direct electrical contacts between the Bi_2_Se_3_ nanostructures and the MWCNTs (Figure 1c,d), resulting in effective charge transfer between the Bi_2_Se_3_ and MWCNT, contributing to the overall electrical conductivity of the composite.

Comparison of the calculated thermoelectric power factors (PF) of PVOH/Bi_2_Se_3_-MWCNT studied in this work with the properties reported by Pires et al. [16] of PVOH/Bi_2_Te_3_ composites showed that despite the higher the Seebeck coefficient of Bi_2_Te_3_ in comparison with Bi_2_Se_3_, the PF of PVOH/Bi_2_Se_3_-MWCNT composites with 15 wt.% and 30 wt.% of filler (0.7 nW·m^−1^·K^−2^ and 2.3 nW·m^−1^·K^−2^) are ~7–70 times higher than the PF of PVOH/Bi_2_Te_3_ with the 60–75 wt.% filler (0.01–0.3 nW·m^−1^·K^−2^), Figure 2c, Table 1.

Therefore, the fabricated n-type PVOH/Bi_2_Se_3_-MWCNT composites show thermoelectric properties significantly higher in comparison to the state-of-the-art PVOH/Bi_2_Te_3_ using three times less wt.% of the n-type inorganic particles and employing less-toxic materials. Presumably, such efficiency of the PVOH/Bi_2_Se_3_-MWCNT composites is due to the electrically conductive network formed by MWCNTs and due to direct electrical contact between the Bi_2_Se_3_ nanostructures and MWCNTs, resulting in effective charge transfer between the Bi_2_Se_3_ and CNTs, significantly contributing to the electrical conductivity of the composites. In addition, the MWCNT network improves mechanical stability and flexibility of the PVOH/Bi_2_Se_3_-MWCNT composites.

**Figure 3:**
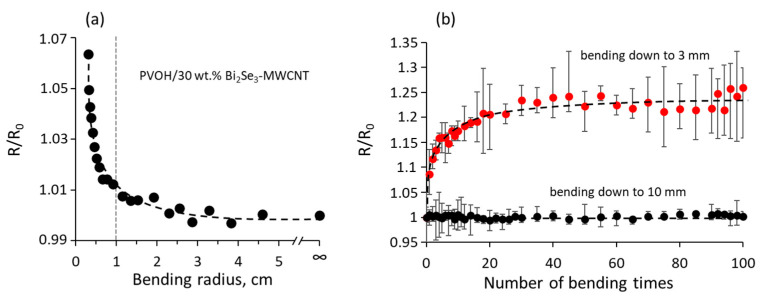
(**a**) Relative changes in the resistance of the PVOH/Bi_2_Se_3_-MWCNT composite with 30 wt.% Bi_2_Se_3_-MWCNT filler vs. its bending radius (R is the resistance of the bent composite, R_0_ is the resistance of the composite before the first bending cycle); (**b**) relative changes of the resistance of PVOH/Bi_2_Se_3_-MWCNT composite with 30 wt.% Bi_2_Se_3_-MWCNT filler under repetitive bending down to 10 mm (black dots) and 3 mm (red dots) vs. bending radius.

The bending tests performed at room temperature have shown that the change of the resistance of PVOH/Bi_2_Se_3_-MWCNT composite with 30 wt.% Bi_2_Se_3_-MWCNT filler during the first bending cycle down to 10 mm radius is negligible (less than 1%, Figure 3a), and the resistance of the composite remains constant under 100 repetitive bending down to 10 mm (Figure 3b, black dots). These results suggest that the total contact area and/or number of the nanocontacts between the Bi_2_Se_3_-MWCNT network components inside the polymer reversibly decrease upon bending and recover when the composite is straightened.

In turn, bending down to the radius of 3 mm results in the increase of the resistance by ~6.5% during the first bending cycle (Figure 3a). The repetitive bending of the PVOH/Bi_2_Se_3_-MWCNT composite with 30 wt.% Bi_2_Se_3_-MWCNT filler results in an increase of its resistance by ~25% during the first 25 bending cycles, followed by the resistance stabilization (Figure 3b, red dots). Presumably, bending of the composite down to 3 mm results in partially reversible separation of the nanostructures in the Bi_2_Se_3_-MWCNT networks inside the polymer. However, despite the initial increase of the resistance of the composite, its further stabilization after 25 bending cycles indicates good mechanical durability of the PVOH/Bi_2_Se_3_-MWCNT composites.

In addition, study of influence of Bi_2_Se_3_-MWCNT filler on important for applications of polymer-based composites as temperature-induced expansion/contraction behaviour, thermal stability and temperature dependence of storage modulus was performed.

**Figure 4:**
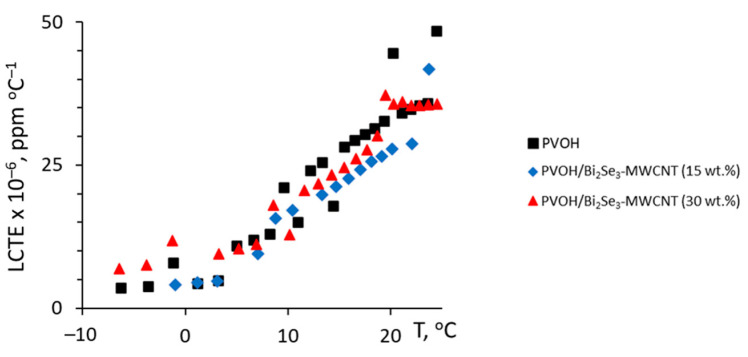
Linear coefficient of thermal expansion (LCTE) vs. temperature of neat PVOH and PVOH/Bi_2_Se_3_-MWCNT composites.

It was found that the linear coefficient of thermal expansion (LCTE) of the PVOH/Bi_2_Se_3_-MWCNT with 15% filler was slightly lower than the LCTE of the neat PVOH (Figure 4, blue squares), presumably due to the known effect of improvement of dimensional stability of polymers by the addition of nanofillers and restriction of macromolecular movement [32].

However, the LCTE of the PVOH/Bi_2_Se_3_-MWCNT composite with 30 wt.% of filler was close to that of neat polymer matrix (Figure 4), presumably, due to the partial agglomeration of the filler.

**Figure 5:**
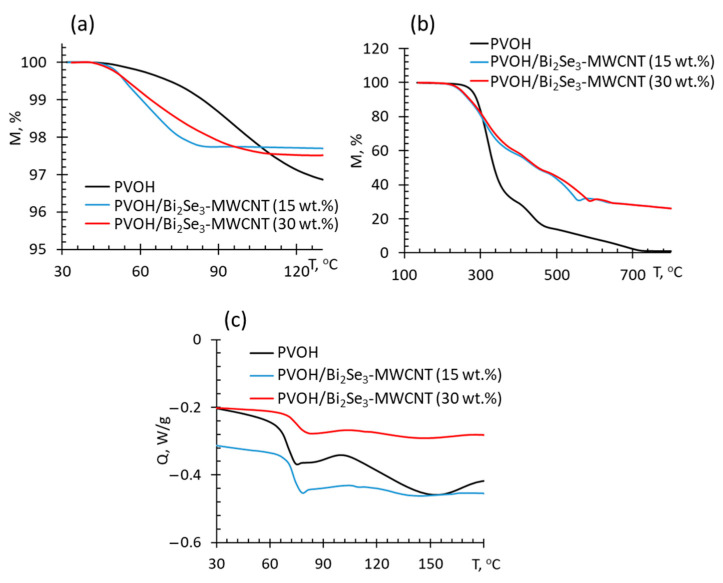
Mass loss of PVOH based thermoelectric composites vs. temperature relationships, recorded at oxidative environment within temperature diapason between (**a**) 30–130 °C and (**b**) 130–700 °C as well as differential scanning calorimetry thermograms obtained after the removal of thermal history of the samples (second run of measurements) (**c**) of various PVOH-based composites.

Thermogravimetric (TGA) analysis of the neat PVOH and the prepared PVOH/Bi_2_Se_3_-MWCNT composites revealed that the Bi_2_Se_3_-MWCNT filler significantly contributes to the moisture (Figure 5a) and thermal barrier properties of the composites (Figure 5b).

At temperatures between 40 °C and 130 °C mass loss of the investigated composites is attributed to the evaporation of moisture as detected by differential scanning calorimetry (Figure 5c). It is important to mention that, by addition of Bi_2_Se_3_-MWCNT, the moisture evaporation peak is decreased, denoting to improved barrier properties of the composites. By considering that moisture may significantly affect electrical and thermal conductivity and hence thermoelectric behaviour of the material, it is believed that upon introduction of the Bi_2_Se_3_-MWCNT thermoelectric fillers in both concentrations investigated, thermoelectric performance of the PVOH composite will be more stable and less dependent on the fluctuations of external moisture content. At temperatures above 220 °C the mass loss of PVOH is observed at ~250 °C, i.e., close to the melting of the crystalline phase of the polymer. Decomposition of the PVOH/Bi_2_Se_3_-MWCNT composites starts at ~220 °C (Figure 5b), which is consistent with the TGA of PVOH/CNT [33] and PVOH+Fe [34] composites and presumably is related to the presence of residues of iron catalyst in the composites, resulting in lowering of their melting temperature.

**Figure 6:**
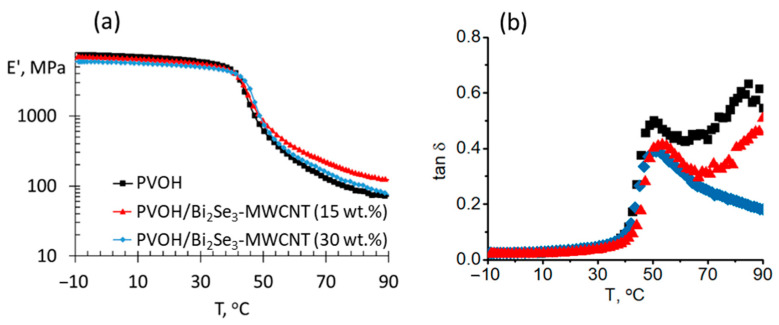
(**a**) Storage modulus and (**b**) tan δ vs. temperature relationships of various PVOH-based composites.

Within the temperature range of 300–700 °C, thermal stability of the PVOH/Bi_2_Se_3_-MWCNT composites is higher than that for neat PVOH, which is consistent with the reports of the other research groups on thermal decomposition of the PVOH-based composites with different fillers as CNTs and inorganic salts [33,34,35], and may be related to the better heat transfer properties through the Bi_2_Se_3_-MWCNT hybrid network, as well as the shielding effect of the fillers, limiting gas exchange in the zone of burning, and possibly also the oxidation of Bi_2_Se_3_, resulting in the formation of a refractory Bi_2_O_3_ oxide layer covering the Bi_2_Se_3_ nanostructures and MWCNTs. At the same time, the difference in thermal stability of PVOH/Bi_2_Se_3_-MWCNT composites with 15 wt.% and 30 wt.% filler was negligible, possibly due to the partial agglomeration of the filler in the composite with 30 wt.%, which is supported also by the conclusions drawn from the LCTE analysis (Figure 4), as well as by the values of the storage modulus of the PVOH/Bi_2_Se_3_-MWCNT composites (Figure 6a). Storage modulus of the composite with 15 wt.% filler (Figure 6a, red dots) in the temperature region above the glass transition temperature is noticeably higher in comparison with the neat PVOH (Figure 6a, black dots).

However, in the case of the introduction of 30 wt.% of Bi_2_Se_3_-MWCNT in the PVOH (Figure 6a, blue dots), only an insignificant increase of the storage modulus is observed in the temperature region above the glass transition temperature in comparison with the neat PVOH, which indirectly supports the hypothesis about the partial agglomeration of the filler. T_g_ determined from tan δ peaks (Figure 6b) show the increase of T_g_ from 50.4 °C (PVOH) to 51.3 °C and 53.5 °C for PVOH/Bi_2_Se_3_-MWCNT composites with 30 wt.% and 15 wt.% filler contents due to stiffening effect of the thermoelectric fillers. These results show that the maximum operating temperature for these composites in thermoelectric applications should not exceed 40 °C to maintain mechanical strength, which indicates the suitability of the developed materials for application in wearable thermoelectric devices.

## 4. Conclusions

PVOH/Bi_2_Se_3_-MWCNT flexible thermoelectric composites with 15 wt.% and 30 wt.% filler were fabricated by mechanical mixing of PVOH with innovative Bi_2_Se_3_-MWCNT hybrid structures having a ratio of 90 wt.% Bi_2_Se_3_:10 wt.% MWCNT, prepared by direct growth of Bi_2_Se_3_ nanostructures on the surfaces of MWCNTs.

It was shown that introduction of the 30 wt.% Bi_2_Se_3_-MWCNT (Bi_2_Se_3_ 90 wt.% and MWCNT 10 wt.%) filler into the PVOH results in n-type PVOH/Bi_2_Se_3_-MWCNT composite, showing a thermoelectric power factor ~7 times higher in comparison with the power factor of state-of-the-art PVOH/Bi_2_Te_3_ composites with 75 wt.% of inorganic Bi_2_Te_3_ filler due to a Seebeck coefficient comparable with that of the inorganic nanostructured Bi_2_Se_3_ material, and electrical conductivity 1–3 orders of magnitude higher than that of PVOH/Bi_2_Te_3_ counterparts. Presumably, such a high efficiency of Bi_2_Se_3_-MWCNT filler could be attributed to the formation of electrically conductive MWCNT networks inside the PVOH, contributing to the electrical conductivity of the composites, and by the direct electrical contact between the Bi_2_Se_3_ and MWCNT surfaces, inducing the charge transfer between the MWCNTs and Bi_2_Se_3_ and significantly contributing to the total value of the Seebeck coefficient of the composites. The mechanical flexibility tests showed stability of the resistance of the PVOH/Bi_2_Se_3_-MWCNT composite with 30 wt.% Bi_2_Se_3_-MWCNT filler during 100 cycles of bending down to 10 mm radius, and a 25% increase of the resistance during the first 25 bending cycles followed by its stabilization when the composite is bent down to the radius of 3 mm.

Additionally, fabricated n-type PVOH/Bi_2_Se_3_-MWCNT composites revealed similar higher thermal stability in the temperature region 300–700 °C in comparison with the neat PVOH. However, the values of the linear coefficient of thermal expansion and storage modulus of the PVOH/Bi_2_Se_3_-MWCNT composite with 30 wt.% indicated partial agglomeration of the filler.

The addition of Bi_2_Se_3_-MWCNT filler to the PVOH also resulted in improvement of the barrier properties of the composites, making them more stable and less dependent on the fluctuations of external moisture content in comparison with the neat PVOH. These results indicate the high potential of the demonstrated PVOH/Bi_2_Se_3_-MWCNT composites for application in wearable flexible thermoelectrics. However, for practical applications as n-type component in thermoelectric generators, the PVOH/Bi_2_Se_3_-MWCNT composites should be improved by development of a method for the increase of filler concentration in the composite without agglomeration.

## Figures and Tables

**Table 1:** Comparison of properties of PVOH-based composites with Bi_2_Se_3_-MWCNT and Bi_2_Te_3_ fillers in different concentrations.

Filler Type	Filler wt.%	ρ, Ω·m	S, μV·K^−1^	PF, nW·m^−1^·K^−2^	Reported
Bi_2_Se_3_-MWCNT	10	>1 × 10^9^	at the noise level	~0	This work
Bi_2_Se_3_-MWCNT	15	11.5 ± 0.5	−60 ± 5	0.28 ± 0.04	This work
Bi_2_Se_3_-MWCNT	30	4.8 ± 1.5	−100 ± 4	2.5 ± 1.1	This work
Bi_2_Te_3_	68	3333	−175	0.01	Pires et al. [16]
Bi_2_Te_3_	75	100	−173	0.3	Pires et al. [16]
Bi_2_Te_3_	90	1.25	−170	23.12	Pires et al. [16]
Reference bulk materials					
Bulk Bi_2_Te_3_	-	-	~−68	-	Navrátil et al. [25]
Bulk Bi_2_Te_3_	-	-	−225	-	Witting et al. [26]

## Data Availability

The data presented in this study are available on request from the corresponding author. The data are not publicly available as they are part of ongoing research.
